# Supplementary figures and images for: How does reorganisation in child and adolescent mental health services affect access to services? An observational study of two services in England
Source: PLoS One. 2021 May 5;16(5):e0250691. doi: 10.1371/journal.pone.0250691 (PMC8099077; doi:10.1371/journal.pone.0250691)

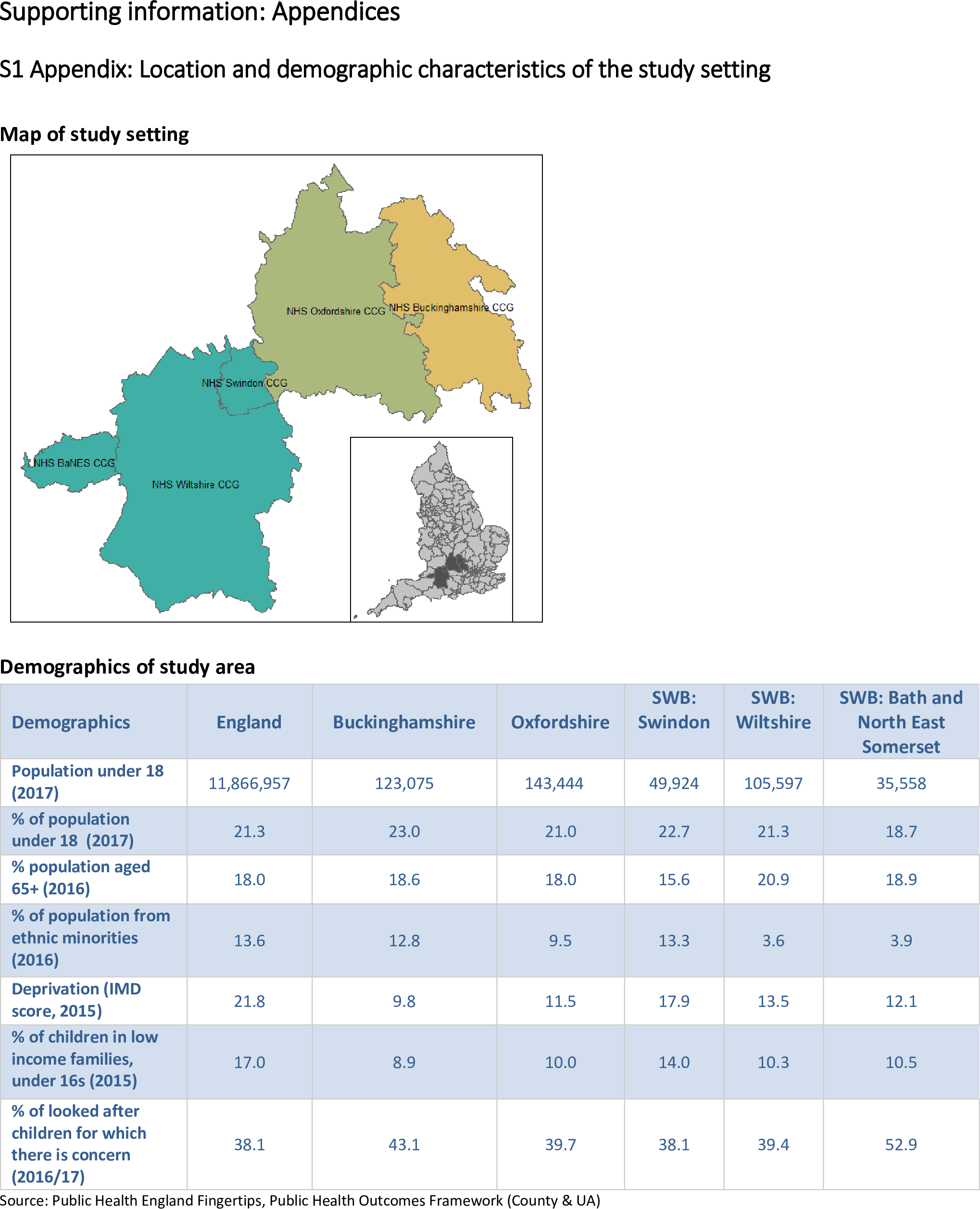

Supplement: S1 Appendix — (TIF) [file pone.0250691.s001.tif]

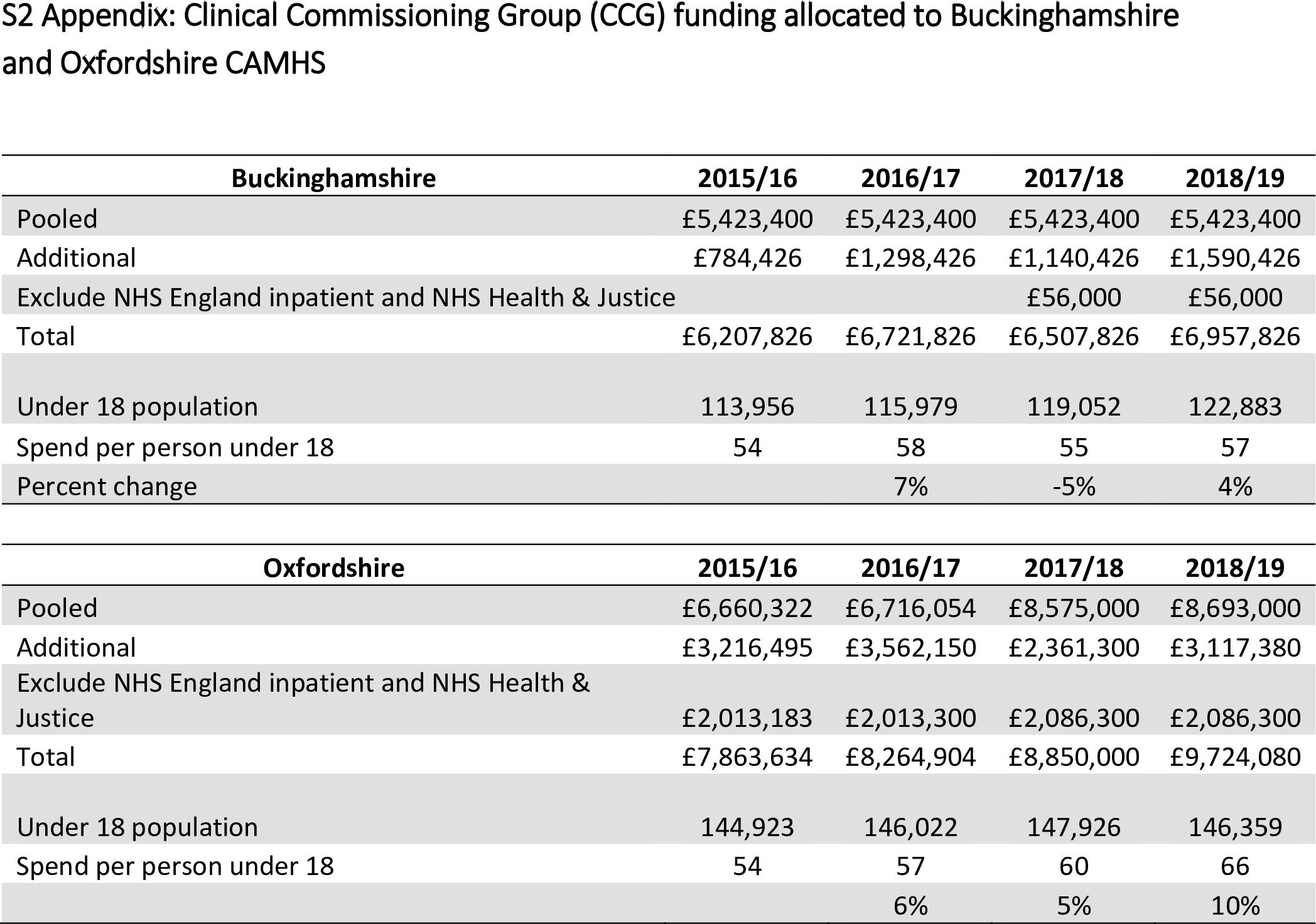

Supplement: S2 Appendix — (TIF) [file pone.0250691.s002.tif]

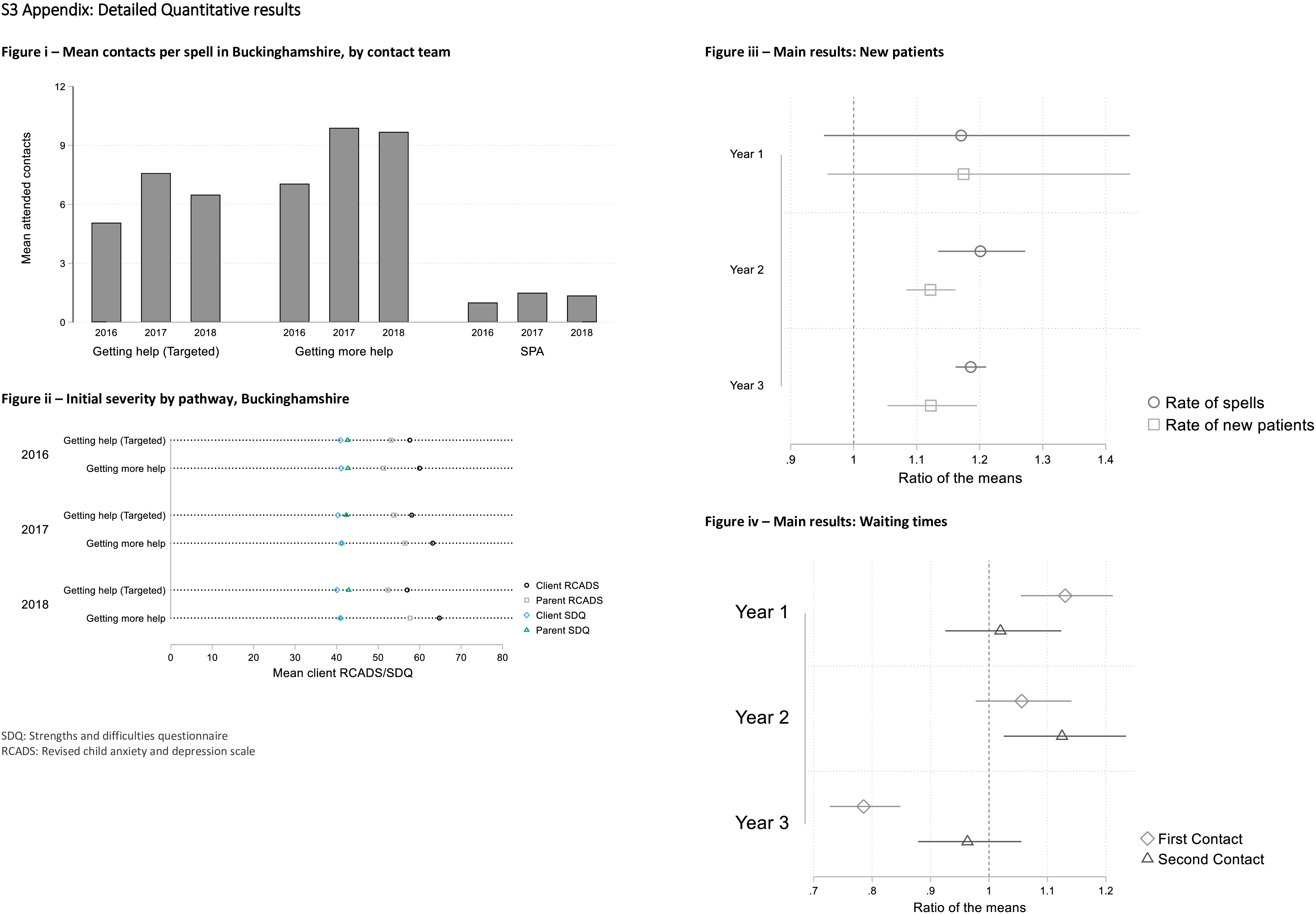

Supplement: S3 Appendix — (TIF) [file pone.0250691.s003.tif]
