# Supplementary material for: How does reorganisation in child and adolescent mental health services affect access to services? An observational study of two services in England
Source: PLoS One. 2021 May 5;16(5):e0250691. doi: 10.1371/journal.pone.0250691 (PMC8099077; doi:10.1371/journal.pone.0250691)
Supplement: Attachment — Submitted filename: Response to Reviewers - PONE-D-20-38869R1.docx [file pone.0250691.s005.docx]

Detailed Response to Reviewers comments:

1. Please ensure that your manuscript meets PLOS ONE's style requirements, including those for file naming. **DONE**

2. Data Availability:

We note that you have indicated that data from this study are available upon request. PLOS only allows data to be available upon request if there are legal or ethical restrictions on sharing data publicly. For information on unacceptable data access restrictions, please see http://journals.plos.org/plosone/s/data-availability#loc-unacceptable-data-access-restrictions.

a) If there are ethical or legal restrictions on sharing a de-identified data set, please explain them in detail (e.g., data contain potentially identifying or sensitive patient information) and who has imposed them (e.g., an ethics committee). Please also provide contact information for a data access committee, ethics committee, or other institutional body to which data requests may be sent.

We will update your Data Availability statement on your behalf to reflect the information you provide.

**Done**: we have updated this so that it now reads:

### *Data sharing statement*

*Raw data is sensitive patient data from Oxford Health NHS Foundation Trust and can be provided on direct application from Oxford Health NHS Foundation Trust to the following email:* [*cris.admin@oxfordhealth.nhs.uk*](mailto:cris.admin@oxfordhealth.nhs.uk) *and by following the procedures as outlined in the following website: https://www.oxfordhealth.nhs.uk/research/toolkit/cris/. The study data was accessed as part of a data sharing agreement with the University of Oxford.*

3. Your ethics statement should only appear in the Methods section of your manuscript. If your ethics statement is written in any section besides the Methods, please move it to the Methods section and delete it from any other section. Please ensure that your ethics statement is included in your manuscript, as the ethics statement entered into the online submission form will not be published alongside your manuscript.

**Done**. This has now been moved to the appropriate place.

4. We note that Figure S1 in your submission contain map images which may be copyrighted. All PLOScontent is published under the Creative Commons Attribution License (CC BY 4.0), which means that the manuscript, images, and Supporting Information files will be freely available online, and any third party is permitted to access, download, copy, distribute, and use these materials in any way, even commercially, with proper attribution. For these reasons, we cannot publish previously copyrighted maps or satellite images created using proprietary data, such as Google software (Google Maps, Street View, and Earth). For more information, see our copyright guidelines: http://journals.plos.org/plosone/s/licenses-and-copyright.

 We require you to either (1) present written permission from the copyright holder to publish these figures specifically under the CC BY 4.0 license, or (2) remove the figures from your submission:

 4.1.    You may seek permission from the original copyright holder of Figure S1 to publish the content specifically under the CC BY 4.0 license.

**Done**. The maps are available under an [open licence agreement](https://www.ons.gov.uk/methodology/geography/licences), which is [compatible](http://www.nationalarchives.gov.uk/doc/open-government-licence/version/3/) with the creative commons licence. We have also added the following to the figure:

*Source: Office for National Statistics licensed under the Open Government Licence v.3.0
Contains OS data © Crown copyright and database right 2018*

 5. Please include captions for your Supporting Information files at the end of your manuscript, and update any in-text citations to match accordingly. Please see our Supporting Information guidelines for more information: [http://journals.plos.org/plosone/s/supporting-information](about:blank).

**Done**.

6. We noticed you have some minor occurrence of overlapping text with the following previous publication(s), which needs to be addressed:

https://bmchealthservres.biomedcentral.com/articles/10.1186/s12913-020-05463-4
https://bmjopen.bmj.com/content/bmjopen/10/1/e034067.full.pdf
https://bmjopen.bmj.com/content/10/1/e034067.full

**Done.** References 16, 17, 21 and 25 have now been cleaned (original numbers).

7. Upload your figure files to the Preflight Analysis and Conversion Engine (PACE) digital diagnostic tool,

**Done**

8. Have the authors made all data underlying the findings in their manuscript fully available?

The [PLOS Data policy](http://www.plosone.org/static/policies.action#sharing) requires authors to make all data underlying the findings described in their manuscript fully available without restriction, with rare exception (please refer to the Data Availability Statement in the manuscript PDF file). The data should be provided as part of the manuscript or its supporting information, or deposited to a public repository. For example, in addition to summary statistics, the data points behind means, medians and variance measures should be available. If there are restrictions on publicly sharing data—e.g. participant privacy or use of data from a third party—those must be specified.

Reviewer #1: No

***Clarification****: we have explained that the data that we have used for this analysis is from the Electronic Patient Record held by Oxford Health NHS Foundation Trust. It is therefore highly sensitive and identifiable patient information therefore, according to the data sharing agreement, this data can be requested directly from Oxford Health NHS Foundation Trust. Applications to access the data can be made directly to the following email:* [*cris.admin@oxfordhealth.nhs.uk*](mailto:cris.admin@oxfordhealth.nhs.uk) *and follow the procedures as outlined in the following website: https://www.oxfordhealth.nhs.uk/research/toolkit/cris/*

Review Comments to the Author

Reviewer #1: Thank you for the opportunity to review this manuscript. Ways to evaluate the impact of changes in health services have not been fully addressed in the literature, so this article could make important contribution to the field. However, before it gets published, I would like to suggest authors address following comments, especially the way they organized the introduction and presented the results, to increase the likelihood of having meaningful contribution to the field.

***Thank you****. We appreciate the time you have put to helping us make this study, which we believe provides essential data on how broad service changes can be evaluated, of greater use to the research and clinical community. We hope the changes we have made are satisfactory.*

Overall suggestions:
1. Please define the terms used in the manuscript clearly. For example, how authors define “access to service”? Similar issues with “delivery pathway”, “tiered service”, and “third sector partners”. For readers who are not familiar with services in UK. It would be helpful to know the definitions of these terms.

**Done**: we have addressed this throughout and tried to clarify the terms utilised, for example with Third Sector we have added *‘(charity, social enterprises and voluntary group) partners’* at its first mention in the text and have added a description of the ‘Tiered’ model of service delivery.

2. Please check acronyms (e.g., CAHMS, SPA, CCG) used in the manuscript, including figures and tables, to make sure they are spelled out and explained what they mean the first time they show up, and then use acronyms thoroughly afterwards. For example, CAMHS was first spelled out in p.4 instead of p.3. Authors sometime used the full name again after the acronyms has been introduced (e.g., line 325, p.15).

***Done****: we apologise for this and have now endeavoured to ensure all acronyms, where used, are clarified. We have removed ‘CYP’ entirely, removed WTE from the text, and as for CCG we have decided to spell this out in the 3 places it is mentioned so as to reduce the memory load, but maintain the ‘(CCG)’ after they are first mentioned, as this is how these groups are referred to in the UK.*

3. Please use terms consistently throughout the manuscript. For example, children and young people (CYP) or adolescent? Main analysis or primary analysis?

***Done****: we agree that this needs to be consistent and apologise. We have now tried to use: ‘children and adolescents’ throughout with very occasional use of ‘young people’ and removal of CYP.*

INTRODUCTION
Overall, the links between each paragraph in the introduction were not clear, and the justification of conducting this study was not clearly elaborated. Authors described the aim of this study in the end of the third paragraph. However, the overall knowledge gap was not clearly described and summarized, so the justification of conducting this study was not very compelling in the introduction. I suggest authors reorganize the introduction to summarize the knowledge gap in a more comprehensive way, and make the link between the knowledge gap and the aim of this study more explicit. Questions regarding each paragraph were listed below:

**Done**: In light of the many helpful comments about the Introduction here and below, the introduction has been completely rewritten, with the scope narrowed, one reference added and clarification provided on what we mean when examining ‘access to services’. The new introduction reads as follows:

*Concerns across high-income nations over the adequacy of healthcare services to meet the needs of children and adolescents [1, 2] is propelling services across a number of nations to institute broad systemic change as insufficient numbers are actually being seen by services. Making child and adolescent mental health services (CAMHS) more accessible to young people is driving many changes taking place in England, which have been mirrored in countries including Canada, Australia, the US and Ireland [3-8]. Access to services for children and adolescents include a number of different factors, including: knowledge, attitudes, and beliefs about mental health problems and treatment held by both young people and their parents; where these services are geographically located; how they can be approached; and once approached whether they have the resources to address any identified need [9].*

*In England the focus on improving access to services, for those under the age of 18, has led to a major reorganization of CAMHS aligned with the THRIVE model which recommends principles by which services can be organized [10] emphasising five areas: Thriving, Getting advice and signposting, Getting Help, Getting More Help and Getting Risk Support. The focus of this model lies in making the experience of mental health services for the young person less confusing and encourages shared decision-making with the children, adolescents and their families. It tries to ensure that all the agencies involved in a young person’s life work together in as coherent and integrated a manner as possible.*

*Government directives have therefore encouraged the many CAMHS services via commissioners to ‘transform’ their services to improve accessibility, increase quality of care and improve health outcomes [4, 11], although the pace and direction of change is determined at the local level. Therefore a natural experiment is taking place across CAMHS in England where different components of services are being changed to try and better address the identified needs of the young population and improve access to services in general. Many of the services have even adopted the THRIVE terms for the different parts of their newly ‘transformed’ services.*

*This observational mixed methods study aims to build on the limited evidence-base that currently exists on the impact and implementation of these system wide changes to CAMHS. We will describe components of two CAMHS services experiencing service ‘transformation’ at different time points and with some differences in how the changes are translated and evaluate their resultant changes in access to services, as measured by the actual number of children and adolescents entering the services.*

1) In the 1st paragraph, authors described that major changes have been taking place in the organizations of mental health services, the cost and prevalence related to mental health in UK, but I was left wondering what the current unmet needs were or what the service gap looked like in UK.

***Done****: as described above we have rewritten the Introduction and tried to place emphasis on these points.*

2) The 2nd paragraph included concerns across high-income nations regarding adequacy of healthcare services and listed recommendations regarding how services should meet the needs of the most vulnerable. However, it was unclear to me how these concerns and recommendation were linked to the mental health services specifically. If authors tried to use this paragraph to highlight the importance of improving access, they need to make it more explicit for readers.

***Done****: as described above in the major reorganisation and change in emphasis in the Introduction.*

3) The 3rd paragraph talked about many CAMHS transferred from traditional tiered model to integrated service informed by the THRIVE model. However, it was unclear to me what existing challenges with the traditional tiered model were, how changing to integrated services could address current unmet needs, and why authors wanted to highlight THRIVE model specifically? Was it the most commonly used model across services? Please clarify.

***Clarified****: this is harder to address than one would think, as the THRIVE model has been identified by central government rather than by services, and the evidence-base on which is has been promoted is unclear, hence the rationale for conducting this study. We hope with the newly written introduction, this is made clearer.*

4) The 4th paragraph talked about the evaluation of impacts and implementation of changes remained limited, the government had recommendations to ‘transform’ services, and there was significant investment in local Transformation Plans. It was unclear to me what the main idea of this paragraph was. What unmet needs caused the government to argue there’s a need to transform service? What exactly did the local transformation plans look like, and how long have these plans been implemented? What problems would exist if we didn’t evaluate impacts of changes?

***Done****: as described above in the major reorganisation and change in emphasis in the Introduction.*

5) The 5th paragraph talked about several strategies to improve services. However, it was unclear what authors’ interpretations regarding previous literature were and how these previous findings were linked to this study.

***Done****: as described above in the major reorganisation and change in emphasis in the Introduction we have removed much of this section.*

6) The 6th paragraph talked about a WHO review highlighted the importance of improving mental services and having consistent way to collect and report data. However, I don’t see how the main idea of this paragraph was connected to other paragraphs in the introduction. I would suggest authors to move it to the discussion (e.g., line 335-337, p.15).

***Done****: we agree, and have removed much of this section.*

METHODS
1. Study setting: Please provide more background information about how service transformations were taking place in Bucks and Oxon. For example, when the transformation started in each area? What were the goals of transformation in each area? Possible reasons why these two areas had service transformations while other areas did not?

***Done:*** *much of this is described in the study protocol, but we agree that some more context is needed for the reader. Thank you for this suggestion. We have added the following to the Study Setting Section in the Methods.*

*We investigated the CAMHS changes or ‘transformation’ in Bucks and Oxon. These services are commissioned locally and therefore we were able to examine the changes taking place in the different areas as described in the study protocol [13]. The changes in Bucks took place from 2015 and in Oxon from 2018 as each area made changes according to when their service agreements are being renegotiated and designed. For many services these take place on a five-year cycle and therefore depending on when these are confirmed, the new services were introduced.*

2. Overall, there was not enough detail regarding qualitative data and analysis. Authors only listed types of qualitative data they collected, including observations and interviews with stakeholders. It would be helpful for readers to know the focus of observations and guiding questions they had for interviews. In addition, there was only one sentence about data analysis, which did not provide enough information for readers to evaluate the rigor of the data analysis process.

**Done**. Following previous comments we had removed much of this but we agree and welcome an opportunity to re-add this. For the in-depth interviews, we have included the semi-structured guide used at the end of this document and have summarised this in the text. We are happy to add this guide as an appendix if needed.

The new text in the qualitative data analysis section is as follows:

*Observations were determined by access to particular settings (such as the SPA and meetings) through key staff or ‘gatekeepers’. A field diary was kept throughout to record observations with particular attention paid to the RE-AIM framework. The objective was to record and observe naturally occurring interactions: watching, listening, and better understanding how transformation processes were occurring and being interpreted ‘on the ground’. The observation period for this study included both a pre- and post-transformation period. In addition in depth interviews …..The questions asked were framed around the implementation questions of interest, including what they perceived as the main changes taking place in their organisations, what they thought the benefits and problems were associated with these changes and the processes by which these changes had been introduced and sustained*

3. Line 155-159, p.7: It would be helpful for readers to see the strengths of having a mixed methods design if authors could provide several examples to elaborate how they integrated QN and QL data to achieve a more complete overall interpretation.

Done: We have also added the following in the Quantitative data and analysis section which also addresses some of the questions asked in the previous comment:

*We adopted a parallel convergent mixed methods design [19]. The quantitative and qualitative data were collected concurrently and analysed separately. We then integrated the quantitative and qualitative datasets to address the research questions in the following way. Hypotheses (arising from the quantitative data) and themes (from the qualitative data), generated from the separate analyses, were explored in the light of the findings from the other dataset. In this way, key issues emerging from one of the components was then examined alongside evidence from the other component. This enabled us to obtain explanation and context around an issue, as well as prompting new questions to take back to the other dataset. This approach, known as ‘following a thread’ (described by O’Cathain et al) [20], which involves iteratively moving between quantitative and qualitative datasets, enabled us to achieve multiple perspectives on the transformation and more complete interpretation and understanding of the impacts of the reorganization.*

RESULTS
1. As authors described in the method section, there were 5 research questions. It would be easier for readers to follow if authors could consider revising the sub-headings of the result section to make the link between results and each research question more explicit.

**Done**: there are three main areas we will now focus on and these three are separately reported in the results section. We have removed the other areas which take the focus away from the main thrust of the paper. We previously reported on a more complete picture but as a result of previous peer review at PLOS One, the encouragement was to move to a more refined report and therefore we would be concerned that broadening it again is not aligned with the overall, sometimes conflicting requests:

- *a single point of access (SPA) to CAMHS with the possibility for self-referral;*
- *new service delivery pathways and other specialist teams;*
- *third sector partners directly commissioned to deliver services with or as part of CAMHS*

Across different parts of the result section, there were two main issues needed to be further addressed, as described below:
1) First, sometimes it was unclear for readers to see how the findings were well supported by what kinds of data. For example, in p.7, authors summarize that Bucks and Oxon had similar core components of transformation, but as a reader, it was unclear to me whether these findings were supported by any of the QN or QL data authors collected.

**Done**: this is now clarified

Similarly, in p.8-9, authors described that the introduction of the SPA was perceived by staff as the core change of the CAMHS transformation, but it was unclear to me whether this finding was supported by interviews with staff OR it was supported by observations OR both. Authors also described the numbers of people accessing SPA climbed quickly without providing related data to support this finding.

**Done**: Both of these is correct and this has now been clarified and referenced. The following has been added to the section on SPA:

*This was evident from the redesign of how the services were being managed, the staff interviews and the observations from the services.*

2) Second, the service transformations were taking place in both Bucks and Oxon. However, sometimes authors only described findings of one area and did not provide further explanations regarding another. I understand that these two areas started service transformations at different time, so it makes sense that some findings can only be illustrated by data of Bucks. However, I still think authors should at least have several sentences to address this issue as a whole, maybe in the method section or in the beginning of the result section to act as an orientation for readers. In addition, in the facilitators and barriers section, it was also unclear to me whether the findings were support by data of two areas or only by data of one area.

**Done**. As above, we have now clarified the different roll-out of the changes in the different areas. We have also provided further clarifications in the facilitators and barriers section, for which the learning is across both sites and in order to protect the privacy of those interviewed have not disaggregated the results if this is not supported by the findings.

2. Other suggestions:
1) Line 241-242, p.11: It seems that the “tension” is an important finding, since authors decided to have it in the abstract and the discussion. I suggest authors provide more data to support this finding.

**Clarified**: some additional information is provided however the range of staff responses to really draw on this would change the focus of this paper away from our understanding of what the previous reviewers had requested and back to our previous, original submission (in December 2019). We are grateful for this suggestion though and will bring these components together in another piece of work about the complexities of organisational change which is always of interest in the implementation science world.

2) P. 11-12: The findings regarding facilitators did not seem robust because the statements were not clearly supported by data.
3) P.14, table 1: Regarding the waiting time, it seemed that it increased in year 1, and then decreased in year 3. Was there any other data could provide further explanations?

**Considered**: thank you for raising this important question. There are hypotheses as to why this increased but we did not gather data and so we hope this is pursued in future studies. For example, when changes take place to any ‘boarder’ then those that are trying to access services might be able to benefit from the changes and so referrals can increase until a new equilibrium is reached. This however, is for discussion.

DISCUSSION
Similar to the suggestion above, I suggest authors reconsider the structure of the discussion section to make it more aligned with the research questions they proposed. In addition, the discussion lacked of citing relevant literature to demonstrate how the findings of this study fit with existing knowledge and what new insights they contribute.

**Done**. Thank you, we agree this needed addressing. The discussion has been changed, updated, new references added and shortened.
