# Supplementary material for: How does reorganisation in child and adolescent mental health services affect access to services? An observational study of two services in England
Source: PLoS One. 2021 May 5;16(5):e0250691. doi: 10.1371/journal.pone.0250691 (PMC8099077; doi:10.1371/journal.pone.0250691)
Supplement: Attachment — Submitted filename: Responses to the reviewer comments-resubmission 101220.docx [file pone.0250691.s004.docx]

## New submission: How does reorganisation in child and adolescent mental health services affect access to services? An observational study of two services in England

## Comments on a Previous submission:

Reorganisation and investment in child and adolescent mental health services to improve access: a four-year observational mixed-methods study in England

[**PONE-D-19-33715**].

I attach this working doc here that we as study authors used to address the comments raised, we had not actually planned to submit this along with this paper as we feel this paper is completely different, but as requested, please do review this document with an appreciation of the fact that we feel we have submitted a new paper with a different focus. However the work we have done has been fully informed and shaped by these really thoughtful comments that the reviewers have clearly spent a lot of time thinking about the work and really constructively helping us shape this research, for which we are very grateful.

Introduction:

1. the actual gap between “supply” (i.e., what services can provide now) and “demand” (i.e., the needs of children and young people) was still unclear to the readers:

*DONE: We have addressed all these comments by dramatically shortening the introduction and making it more focused*

2. The topic sentence of the 3rd paragraph was not clearly supported by the information provided in the same paragraph. I suggest authors reorganize the 3rd and 4th paragraph (p. 3 line 68 to p.4 line 81) to make the main ideas of each paragraph more salient.

*DONE: these two paragraphs have been rewritten and in particular, drawing from comments below, have also added further information on the THRIVE model*

3. The authors described several promising strategies to improve services which were identified by previous implementation research, and then they described the findings of previous studies about the ‘Choice and Partnership ‘ Approach. However, it was unclear to me what main points authors tried to argue with this literature review. Were they trying to say previous studies related to examining broad system change only focused on enhancing the engagement of patients and families, but did not address other strategies, which highlight the need to conduct a more comprehensive study to examine broad system change? Please clarify.

*DONE: we agree and drawing from the many comments and the rewriting of the paper, the new focus on just the main outcome of the implementation, we have removed many of these references and kept the focus more tight. Thank you for this.*

Methods:
**1.** Study setting: It seemed that the data from SWB were only used for the main analysis as the control group. Please explain why the authors decided to only investigate the CAMHS transformation in Bucks and Oxon comprehensively, instead of include all areas (i.e., Bucks, Oxon, and SWB).

*DONE- this is now clarified*

**2.** Quantitative data and analysis: p.7 line 152-153, the descriptions of the main analysis and the sensitivity analysis were not aligned with the description of the results and table 1.

*DONE- we have now checked these numbers and removed components of these analyses as we are no longer including them in the new more focused submission*

**3.** One of the strengths of this study is the analysis which combining both qualitative and quantitative data, so I hope authors could provide more concrete examples about how the iterative approach worked for this study.

*DONE- we have taken many of the criticisms of the methodological approach seriously- this is something for which there are many differing views amongst the academic community and so we appreciate that depending on perspectives, approaches are different. We have followed what we describe in our protocol, but for the purposes of this paper, are limiting our emphasis to the main observational findings. We have additionally added a new section on the methodology with a new reference.*

4. The authors did not mention their IRB process.

*This was a service evaluation and although we applied to our Ethical approval board, the were clear that the work we were doing did not fall under their remit as service evaluation does not. We have explained this and also described how we still chose to approach the study following GCP and so for the qualitative interviews, we still gave an information sheet and gathered informed consent. In our paper we describe it as follows:*

*This study was registered with the Oxford Health NHS Foundation Trust*

*Anonymized routinely collected patient level data for this study was shared according to a local data sharing agreement with Oxford Health NHS Foundation Trust. This study was registered with Oxford Health NHS Foundation Trust approved by the Children and Young People’s directorate (4/4/2018). Interview participants gave informed consent for participation.*

Results:
1. Funding for transformation:
(1) Not sure how the spend per head of the target population was calculated. Based on the numbers provided, I can see the spend increased 22% in Oxon, which equals to 66/54, but I don’t know why the spend increased 7% in Bucks. If I understand it correctly, the spend should increase around 6% in Bucks, which is close to 57/54.

*DONE: we have described this now in the text and have adjusted for population and excluded inpatient spend.*

(2) Based on figure 2, it seemed that in Bucks, the “spend per target population” did not continuously increase as time went by, but the proportion of target population with 2 contacts increased steadily. In Oxon, we did not see the similar pattern. However, I did not see any discussion about this finding. Please clarify.

*AGREE: we agree, this relationship is not clear but we have decided to minimise this point and cut the word count by one third and just focus on the main findings. It is still an important point but one for another paper to discuss (if such a paper ever gets written!!! Sadly not by us as this study ended one year ago!)*

2. (1) Please provide some examples about what the authors meant by “awareness-raising activities”.
(2) The authors described how the SPA in Oxon was launched in the beginning, but did not describe the initial situation in Bucks (p.9 line 205-206).

*CHANGED FOCUS: We decided to remove the majority of references to the SPA in this paper and put that in a complementary paper and so we have instead of expanding on the SPA activities, left the focus on this paper the main overall impact of transformation. In a similar vein, we have removed the components of the school InReach service as well as this is more nuanced to Oxfordshire and not as generalisable.*

3. Third sector partnerships: It seemed that the nature of the third sector involvement in Bucks and in Oxon was different. I was left wondering what the pros and cons of collaborating with one third sector partner and with eight different third sector partners. In addition, I only see quotes from staff interviews. Did the target population also share something about their perceptions/experiences interacting with third sector partners?

*CHANGED FOCUS: as above and reduced word count by not making the third sector involvement the core aspect of this paper. This is of relevance as the third sector component in Oxfordshire was just about to be launched.*

4. Improved working with education: Authors mentioned Bucks and Oxon had similar core components of transformation on p.7-8, including closer CAMHS working with schools. However, they only described the situation in Oxon on p.13-14.

*CHANGED FOCUS: as per response to point 2 above.*

5. Facilitators:
(1): Vision for transformation: When I was reading the findings, I felt the facilitator was “staff share the same vision for transformation” instead of just having a vision for transformation. I suggest authors reconsider the name of the subtheme to make it more aligned with the finding.

DONE. Thank you

(2) All the qualitative data were from staff’s perspectives. Were here any data from the target population?

*DONE: we have now added two more quotations from the target population*

6. Barriers:
(1) I think one of the limitations of this study was that the authors were unable to collect perspectives from staff who have left the service during the transformation. However, the perspectives from staff who decided to leave were especially important while discussing barriers of implementing transformation.

*AGREE- We have added this as a limitation.*

(2) It’s important for authors to at least describe a little bit about how the planning and communication around the transformation were implemented before the transformation started, so readers could have a better understanding about how the communication was done while reading the findings. In addition, similar to what I have mentioned above about the name of the subthemes, I also suggest authors revise the name from “communication and leadership” to “lack of communication and leadership” to make it more aligned with the findings.

*DONE*

(3) Regarding challenges around third sector involvement, the authors provided perspectives from staff. Was there any data from the third sector agency staff?

*CHANGED FOCUS: as above and reduced word count by not making the third sector involvement the core aspect of this paper. This is of relevance as the third sector component in Oxfordshire was just about to be launched.*

7. What is the impact of the CAMHS transformation on patient access?
(1) Please spell SA out in Table 1.

*DONE*

(2) The authors only provided quantitative data about the impact of the CAMHS transformation on patient access. Were there qualitative data talking about the patient access as well?

*CHANGED FOCUS: as we have changed the focus of this paper and these patient interfaces were mainly in the SPA, we have reduced our word count here and made this more about the systemic level changes. So we have added some perspectives as described above.*

Discussion:
1. To me, the links between each paragraph in the discussion section were not easy to follow. For example, first, the authors described ideas related to how the transformation took time to show the impacts (p.19 line 414-416), and also provided one-sentence summary about the facilitators and barriers to transformation (p.19 line 416-418). Second, the authors started to talk about self-referrals and how patients were appropriately triaged into the pathways. Third, the authors discussed the facilitators and barriers in a more detailed way (p.19 line 427-p.2020 line 435). Finally, the authors discussed how the transformation took time to show the impacts in a more detailed way (p.20 line 436-453). I felt the main ideas authors tried to argue became less salient and less compelling with the way authors currently organized and presented their ideas.

*DONE: We have tried to simplify this section and also cut the words*

2. I think one of the limitations of this study is that the authors were unable to collect perspectives from staff who left the service during the transformation.

*AGREE- We have added this as a limitation.*

3. In the introduction, when authors described their research questions, the impacts of transformation included not only access issues, but also other aspects of services, such as health outcomes and costs. However, I do not see any related discussions.

*AGREE- This is a more complex area to address and so we have, rather than broaden the scope and the claim of our findings, made it more focused on the issues we can confidently address. WE hope these broader issues are taken up by the other researchers in the field, hopefully drawing on our findings for this.*

Reviewer #2: MAJOR CONCERNS:
- The strength and feature of mixed methods design are to combine and integrate both qualitative and quantitative findings to draw a more comprehensive interpretation/ finding than either quantitative or qualitative alone. However, a study including qualitative and quantitative data but lacking integration of two datasets is not a mixed-methods study. The qualitative data were not integrated with quantitative data by merging, connecting, or embedding in this study, so this study is not qualified for mixed-methods design.

*CHANGED FOCUS: As discussed above, this reviewer clearly has expertise in mixed methodology that is not aligned with the methodology we have decided to adopt. We have therefore taken their critique seriously, but as this is a methodology we have described in our protocol and as we do not believe the approach we have undertaken is seriously flawed, it is difficult to fully address all of these concerns. We do think the main issue is that we have not been sufficiently transparent with what we have done. We have therefore added some more text to clarify what we have done in the methodology section and furthermore, what we have done is focus on this being a simpler, observational study*

*For example some of what we have added is copied here:*

*We adopted a parallel convergent mixed methods design [38]. The quantitative and qualitative data were collected concurrently, analysed separately and then integrated as we used both datasets to address each of the research questions. This approach enabled us to achieve a more complete overall interpretation and understanding of the impact of reorganization.*

- Furthermore, the data for each RQ is not sufficient. Based on the cited study and S2 for study protocol, each RQ was answered by qualitative and quantitative data, except RQ1. However, the results for RQ3 only included qualitative data without quantitative data, and the results for RQ5 only included quantitative data without qualitative data.

*CLARIFICATION: We have tried to answer each question with the approach that we think best highlights the findings related to each question.*

- The results for RQ2 included both datasets, but the two datasets were not integrated or connected. There is no descriptions of how the qualitative data to explain the results from quantitative data, or how the quantitative data support the findings from qualitative data.

*CLARIFICATION: we refer to our response above (the most recent two responses)*

- The qualitative results of RQ2 are more like descriptions of each core component of transformation separately rather than the findings from thematic analysis (the findings from thematic analysis should look like the qualitative results of RQ3, what are the facilitators and barriers to transformation). Using thematic analysis across core components of transformation can be helpful to identify the common characteristics shared by different components and unique characters for each component. The qualitative results of RQ2 should be reorganized.
- The results and discussion of RQ4 was not reported/described in this study.

*CLARIFICATION: we refer to our response above. Fundamental to our understanding is that to determine the more interpretive findings you have to explain/describe the system in the first place. We have cut down on the number of themes presented and tried to say more about these fewer themes. Sometimes qualitative findings can seem more ‘descriptive’ than ‘interpretive’ but nonetheless the qual highlights important points about the components which the quant does not/cannot cover. This paper is not reporting on all of the evaluation but on the main outcomes.*

INTRODUCTION:
1. THRIVE model seems critical to this study, but the information about this model is limited. Please provide more details about this new model and make reader get more understanding about this model (Line 76). E.g., how does this model make person-centered service happen?

*DONE- we have added more text THRIVE in the introduction:*

*Many CAMHS are therefore moving from a traditional ‘tiered’ model where a young person is allocated to a specific tier of service aligned with complexity/need, towards a more ‘integrated’ service, such as that proposed by the THRIVE model – a framework for organizing mental health services around the needs of identified young people and their family’s [12]. THRIVE services are encouraged to focus on five areas for young people’s mental health needs: Thriving, Getting advice and signposting, Getting Help, Getting More Help and Getting Risk Support. Many of the services have even adopted these terms for the different arms of their ‘transformed’ services. THRIVE places emphasis on shared decision-making with the young person and their families, to ensure that the limitations of the evidence-base are understood and that all the agencies involved in a young person’s life work together in as coherent and joined-up a manner as possible.*

2. “two” new CAMHS model (line 80). It made reader confused, since it described (line74-76) “moving from a traditional ‘tiered’ model……, towards a more ‘integrated’ service……” Is the ‘integrated’ service only new one, or are both the ‘tiered’ and ‘integrated’ models new?

*DONE- we have added more text to clarify*

METHODS:
Although a more detailed protocol was described in the cited article, some issues of method are still unclear in either the cited article or this study.
3. Which mixed methods (MM) design was used in this study? Exploratory sequential design/ explanatory sequential design/ convergent design? Please clarify and describe more details about that.

*CLARIFIED: as above with an additional reference added.*

4. Please provide the rationales/ justifications for using MM design in this study.

*CLARIFIED: as above.*

5. Please provide a procedural diagram for MM to present the steps of qualitative and quantitative data collection and analysis. This diagram will help readers to learn the findings come from which dataset and how.

*CLARIFIED: as above, we have made the focus of an observational study.*

6. In mixed methods design study, the RQs typically includes three, one for quantitative data, one for qualitative data, and one for integrated data. Please reconsider the statements of RQs (line 120).

*CHANGED FOCUS: as described above .*

7. For qualitative data collection, the staff including administrative and clinical staff, service managers were recruited. (Did you recruit team leaders, which was described in the cited protocol article, in this study?) (line 131-132) Because the participants with different occupations have different perspectives and work experiences, what was the distribution of the occupations of participants?

*CONSIDERED: we have spent a lot of time trying to address this point, and returned to our data to pull these numbers. Team leaders/managers were recruited but we have concluded that specific roles and a breakdown of these should not be included here or in the paper to protect the identity of staff as they are potentially identifiable from their comments. A range of staff were interviewed across both services that included administrative, clinical staff, service managers and team leaders*

8. Since the duration of observational study was 4 years, and the duration of qualitative data collection was only about 14 months. Have all the staff recruited in this study worked for longer than four years and experienced transformation of services? Please provide more details about the participants.

*CONSIDERED: please refer to the response above. All the staff had experienced transformation as the study draws from data over 4 years, observations were of a much shorter duration. All staff had experienced and/or had views on the service changes because they were currently employed – for the Bucks staff they had a much more retrospective view and for Oxon staff it was new/happening at that time. Staff had been there varying amounts of times and had changed roles within the organisation. Some had been in a variety of roles over many years whilst others had joined more recently i.e. one Bucks staff member was operational lead for one pathway during the service changes but later switched to a different pathway and now does more clinical work. Several Ox staff split time between services but those time allocations and roles were changing… So people’s roles were sometimes quite complex.*

9. Although Swindon, Wiltshire, and Bath and North East Somerset (SWB) was one group included in this study, the staff were recruited only from Oxon and Bucks (line 131). Please provide the justification or add more data to this study.

*DONE: we describe SWB as a control site*

10. The 8 young people and 4 parents were recruited (line 133). What were the diagnoses, severity, and age ranges, and cognitive functions? What were the inclusion criteria for the participants? Please describe it and provide the characteristics of the participants

*CLARIFIED: we have described the inclusion criteria, but as this data above falls under special category data and we agreed not to collect it unless it was absolutely required by the research question. This could then lead to further research questions that might then be more disorder specific but not in the scope of this work.*

11. Please provide more information about Strength and Difficulties Questionnaire and Revised Child Depression and Anxiety Scale (they were not described in the protocol cited article) (line 141-142). The details of scales will help readers understand the measures were used for what and for answering which RQ.

*DONE: we have provided more information on the RCADS but removed all reference to the SDQ as these data were not reported on in this paper (numbers collected too small and to reduce word count).*

12. Please provide more details about distinct spells of care. What distinct spells of care were? What were the criteria for grouping? (line 145)

DONE- clarification provided

13. What statistical tests were used for main analysis (comparison) (line152) and sensitivity analysis (line 153)? Please describe them.

CLARIFICATION- we reference a paper that describes this is substantially more detail and with space constraints we were keen to reduce the overall word count and all this information is available in the referenced publication.

14. ‘Following a thread’ (line 159) is that the findings from the first dataset leads further exploration for the second dataset analysis. However, the description (line 154-159) did not clarify the connection/relationship of the two datasets. If the qualitative data was conducted first, then the key themes/hypothesis were further explored/examined by quantitative data analysis. Please use “qualitative” or “quantitative” dataset in the description, in stead of ‘the other dataset.’ As mentioned earlier, providing a diagram of procedural diagram will help reader understand the whole procedure of data analysis and the connections of two datasets.
15. The definitions of variables that were core components in this study were not described clearly in method. E.g., single point of access, new service delivery pathways, third sector partners, Thrive model, working with school.

*DONE: as referred to in the comments above and paper also simplified as described above.*

RESULTS:
16. There is no descriptions of the study population targeted for study and the inclusion criteria for population should be included method rather than results. i.e., population aged under 18 (line 183), but what was the age range? i.e., common mental disorders (line 183), but what disorders were included for analysis in this study?

*DONE: this is now described in more detail and the appendix 1 gives further information.*

17. The results emphasized young people and parents’ self-referral. If the demographic characteristics of this population, the information will be helpful for the future policy establishment of other institutions based on their own local, unique demographics.

*CHANGED FOCUS: as described above we have removed many of the granular findings about the SPA from this paper and focused on the overall impacts and implementation as a whole.*

18. The sequences and wording of variables listed in figure 1 and descriptions are inconsistent. Please make them consistent to avoid confusion. i.e., Does Thrive model in figure equal to new service delivery pathways and other specialists teams in description (line 165)?

*DONE: thank you for pointing this out.*

19. The study focused on accessibility improvement of mental services, the perspectives of users, the child, adolescent and their parents are critical. If more quotes from the users than one could be provided in this study, the study will be convincing to readers.

*DONE: on review we agree and have reduced half of the quotations from many of the sections.*

20. Please clarify the occupations of staffs cited for the quotes, since the details will help readers understand the perspectives from staff with different responsibility (line 203). E.g., service manager, clinical staff.

CLARIFIED: as described above regarding limitations in collecting sensitive data in the UK.

21. What were the exact numbers of people accessing single point of access (line 204), of requests were received online (line 210), and of increase in self-referrals (line 215)? Please describe it.

*CHANGED FOCUS: as abov, this is no longer the focus of this paper.*

22. “…it took time for necessary space and technology…..to other services or information if CAMHS was not indicated.” (line 205-208). Were the descriptions more likely to present the barriers of accessibility? Please reconsider where the descriptions should be included.

**Any thoughts?**

23. The information of SPA described in New pathways section (line 248) made reader confused. If the description was used to compare, then the description might be moved to discussion part.

*DONE: we have tried to clarify and rationalise throughout.*

24. Is there any results from statistical tests to validate “the initial severity of patients measured by the Revised Child Anxiety and Depression Scale child version was higher among those first entering Getting More Help than Getting Help?” (line 250-252) (the exact numbers and statistics were not present in S4(ii)). Given the comparisons for different years (2016, 2017, 2018) and two service systems, what were the sample sizes, effect sizes, p-values?

*CLARIFIED: we have not been able to conduct this analysis*

25. Please provide information about the content, intensity (duration, frequency, one-on-one or group) of low-intensity interventions provided by buddies (line 258).

*DONE: we have tried to clarify and rationalise throughout and have removed most of the additional components about the third sector and SPA, as described above.*

26. Given the facilitators, how about the self-referral online system for child and young people? What role was played by the technology in mental service transformation? (line 293)

*DONE: we have tried to clarify and rationalise throughout and have removed most of the additional components about the third sector and SPA, as described above. This is however an interesting point but no longer within the scope of the new paper we have now written.*

27. The information from descriptions and table 1 is highly repetitive. Please be concise.

*DONE: we have removed most of the text.*

28. Please removed the irrelevant information from table 1 to avoid making readers confused. E.g., rows for family, link (they should be used for regression analysis rather incidence rate ratio)

*CLARIFICATION: we have checked and confirm that the table presents the relevant findings*

DISCUSSIONS:

29. The problem re-statements should be concise (line 102-413).

*DONE: we agree and have tried to rationalise throughout the whole paper and the discussion section.*

30. The descriptions from line 409-413 are literature review and should be move to introduction.

*DONE: we agree and thank you, some has been moved and some deleted.*

31. The discussion of mixed-methods design should provide descriptions of integration of two datasets and draw more complete and holistic interpretations and findings. However, the integration of results were not discussed or found in this study. The quantitative data was not explained by qualitative and qualitative data was not examined by quantitative data.

*DONE: as described above.*

32. There are a lot of results for RQ2 (SPA, new pathways, third sector, partnerships, and improved working with education) but the results for RQ2 were scarcely discussed. Please provide more discussion about the results for RQ2. E.g., what are the associations between the decrease in percentage of ‘Getting More Help’ (line 228-234) and the changes in distributions of specialists, psychologists, agency staffs, nurses (line 241-243).

*CONSIDERED- We agree these are all very important and interesting questions but we cannot address them here as we have decided to try and make this is more succinct and focused paper, we hope future researchers will be able to scrutinise this.*

33. “The number of attended sessions” (line 425) cannot find the information in results but was reported in discussion. This makes readers confused.

*DONE- we agree and have removed this*

34. Except results for RQ2, the rest results for RQs are either quantitative or qualitative data. The findings seem not to be triangulated (line 460-461).

*DONE: new approach described above.*

FORMATS:
- The abbreviations showed in figures and table should be explained in note below the figures and table. E.g., what did the asterisk * indicate? What did the values in bracket [] and in parentheses () mean?

*DONE: new legend now provided for the table. Thank you for this.*

- The terms and format of quotes (in results) used in this article should be consistent. E.g., THRIVE model; Thrive model.

*DONE*

- The headings in results made reader confused rather than being helpful to understand the study.
o The headings are not the same with research questions (RQs), so might need to be modified.
o In another way, if the 5 (RQs) are used as headings to report the answers/results to the corresponding questions, should the RQ2 as a heading be inserted before heading ‘Funding for transformation’ (line 179)?

*CHANGES MADE: we have tried to rationalise these results and focus on the main findings.*
